# Deaths and Years of Potential Life Lost From Excessive Alcohol Use — United States, 2011–2015

**DOI:** 10.15585/mmwr.mm6930a1

**Published:** 2020-07-31

**Authors:** Marissa B. Esser, Adam Sherk, Yong Liu, Timothy S. Naimi, Timothy Stockwell, Mandy Stahre, Dafna Kanny, Michael Landen, Richard Saitz, Robert D. Brewer

**Affiliations:** ^1^Division of Population Health, National Center for Chronic Disease Prevention and Health Promotion, CDC; ^2^Canadian Institute for Substance Use Research, University of Victoria, British Columbia, Canada; ^3^Boston Medical Center, Boston, Massachusetts; ^4^Boston University Schools of Medicine and Public Health, Boston, Massachusetts; ^5^Forecasting and Research, State of Washington Office of Financial Management; ^6^New Mexico Department of Health.

Excessive alcohol use is a leading cause of preventable death in the United States (*1*) and costs associated with it, such as those from losses in workplace productivity, health care expenditures, and criminal justice, were $249 billion in 2010 (*2*). CDC used the Alcohol-Related Disease Impact (ARDI) application* to estimate national and state average annual alcohol-attributable deaths and years of potential life lost (YPLL) during 2011–2015, including deaths from one’s own excessive drinking (e.g., liver disease) and from others’ drinking (e.g., passengers killed in alcohol-related motor vehicle crashes). This study found an average of 93,296 alcohol-attributable deaths (255 deaths per day) and 2.7 million YPLL (29 years of life lost per death, on average) in the United States each year. Of all alcohol-attributable deaths, 51,078 (54.7%) were caused by chronic conditions, and 52,361 (56.0%) involved adults aged 35–64 years. Age-adjusted alcohol-attributable deaths per 100,000 population ranged from 20.3 in New Jersey and New York to 52.3 in New Mexico. YPLL per 100,000 population ranged from 613.8 in New York to 1,651.7 in New Mexico. Implementation of effective strategies for preventing excessive drinking, including those recommended by the Community Preventive Services Task Force (e.g., increasing alcohol taxes and regulating the number and concentration of alcohol outlets), could reduce alcohol-attributable deaths and YPLL.^†^

CDC has updated the ARDI application, including the causes of alcohol-attributable death, *International Classification of Diseases, Tenth Revision* codes,^§^ and alcohol-attributable fractions.^¶^ CDC used ARDI to estimate the average number of annual national and state alcohol-attributable deaths and YPLL caused by excessive drinking (i.e., deaths from conditions that are 100% alcohol-attributable, acute conditions that involved binge drinking, and chronic conditions that involved medium or high average daily alcohol consumption). ARDI estimates alcohol-attributable deaths by multiplying the total number of deaths (based on vital statistics) with an underlying cause corresponding to any of the 58 alcohol-related conditions in the ARDI application by its alcohol-attributable fraction. Some conditions (e.g., alcoholic liver cirrhosis) are wholly (100%) attributable to alcohol (alcohol-attributable fraction = 1.0), whereas others are partially attributable (alcohol-attributable fraction <1.0) to alcohol (e.g., breast cancer and hypertension). Deaths are assessed by age group and sex and averaged over a 5-year period. The alcohol-attributable fractions for chronic conditions are generally calculated using relative risks from published meta-analyses and the prevalence of low, medium, and high average daily alcohol consumption among U.S. adults, based on data from the Behavioral Risk Factor Surveillance System.** The prevalence estimates are adjusted to account for underreporting of alcohol use during binge drinking episodes (*3*). Alcohol-attributable fractions for acute causes (e.g., injuries) are generally based on studies that measured the proportion of decedents who had a blood alcohol concentration ≥0.10 g/dL (*4*). Alcohol-attributable fractions for motor vehicle crash deaths are based on the proportion of crash deaths that involved a blood alcohol concentration ≥0.08 g/dL.^††^ For 100% alcohol-attributable conditions, deaths are summed without adjustment.^§§^ YPLL, a commonly used measure of premature death, are calculated by multiplying the age-specific and sex-specific alcohol-attributable deaths by the corresponding reduction in years of life potentially remaining for decedents relative to average life expectancies.^¶¶^ Chronic causes of death are calculated for decedents aged ≥20 years, and acute causes are generally calculated for decedents aged ≥15 years. Deaths involving children that were caused by someone else’s drinking (e.g., deaths caused by a pregnant mother’s drinking and passengers killed in alcohol-related motor vehicle crashes) are also included.

CDC used the data available in ARDI to estimate the average annual national and state alcohol-attributable deaths and YPLL associated with excessive drinking and national estimates of alcohol-attributable deaths and YPLL by cause of death, sex, and age group. National and state alcohol-attributable deaths and YPLL per 100,000 population were calculated by dividing the average annual alcohol-attributable death and YPLL estimates, respectively, by average annual population estimates from the U.S. Census for 2011–2015, and then multiplying by 100,000. The alcohol-attributable death rates were then age-adjusted to the 2000 U.S. population.*** The number of YPLL per alcohol-attributable death was calculated by dividing total YPLL by total alcohol-attributable deaths in the United States and in states.

During 2011–2015 in the United States, an average of 93,296 alcohol-attributable deaths occurred, and 2.7 million years of potential life were lost annually (28.8 YPLL per alcohol-attributable death) (Table 1) (Table 2). Among the 93,296 deaths, 51,078 (54.7%) were caused by chronic conditions and 42,218 (45.2%) by acute conditions. Of the 2.7 million YPLL, 1.1 million (41.1%) were because of chronic conditions, and 1.6 million (58.8%) were because of acute conditions. Overall, 66,519 (71.3%) alcohol-attributable deaths and 1.9 million (70.8%) YPLL involved males. Among all alcohol-attributable deaths, 52,361 (56.1%) involved adults aged 35–64 years, 24,766 (26.5%) involved adults aged ≥65, and 13,910 (14.9%) involved young adults aged 20–34 years (Figure).

**TABLE 1:** Average annual number of deaths and years of potential life lost attributable to excessive alcohol use,* by condition and sex — United States, 2011–2015

Cause	Alcohol-attributable deaths			Years of potential life lost		
	Total^†^	Males no. (%)	Females no. (%)	Total^†^	Males no. (%)	Females no. (%)
**Total^†^**	**93,296**	**66,519 (71.3)**	**26,778 (28.7)**	**2,683,211**	**1,899,089 (70.8)**	**784,121 (29.2)**
**Chronic causes**	51,078	35,583 (69.7)	15,495 (30.3)	1,105,190	752,936 (68.1)	352,253 (31.9)
Alcohol abuse	2,591	1,986 (76.6)	605 (23.4)	66,839	49,129 (73.5)	17,710 (26.5)
Alcohol cardiomyopathy	510	432 (84.7)	78 (15.3)	12,235	10,136 (82.8)	2,099 (17.2)
Alcohol dependence syndrome	4,258	3,269 (76.8)	989 (23.2)	109,911	81,192 (73.9)	28,719 (26.1)
Alcohol polyneuropathy	3	3 (100.0)	0 (—)	54	54 (100.0)	0 (—)
Alcoholic gastritis	33	26 (78.8)	7 (21.2)	890	696 (78.2)	194 (21.8)
Alcoholic liver disease	18,164	12,887 (70.9)	5,277 (29.1)	467,996	313,897 (67.1)	154,099 (32.9)
Alcoholic myopathy	0	0 (—)	0 (—)	0	0 (—)	0 (—)
Alcoholic psychosis	703	549 (78.1)	154 (21.9)	14,129	10,799 (76.4)	3,330 (23.6)
Alcohol-induced acute pancreatitis	278	214 (77.0)	64 (23.0)	8,284	6,247 (75.4)	2,037 (24.6)
Alcohol-induced chronic pancreatitis	52	38 (73.1)	14 (26.9)	1,507	1,046 (69.4)	461 (30.6)
Atrial fibrillation	329	228 (69.3)	100 (30.4)	2,943	2,084 (70.8)	860 (29.2)
Cancer, breast (females only)	584	NA	584 (NA)	11,203	NA	11,203 (NA)
Cancer, colorectal	996	898 (90.2)	98 (9.8)	15,540	14,016 (90.2)	1,524 (9.8)
Cancer, esophageal^§^	494	430 (87.0)	64 (13.0)	8,038	7,007 (87.2)	1,031 (12.8)
Cancer, laryngeal	248	233 (94.0)	15 (6.0)	4,002	3,737 (93.4)	265 (6.6)
Cancer, liver	1,609	1,545 (96.0)	64 (4.0)	28,191	27,129 (96.2)	1,061 (3.8)
Cancer, oral cavity and pharyngeal	909	830 (91.3)	79 (8.7)	16,034	14,715 (91.8)	1,319 (8.2)
Cancer, pancreatic^¶^	186	151 (81.2)	35 (18.8)	2,827	2,301 (81.4)	526 (18.6)
Cancer, prostate (males only)	188	188 (NA)	NA	1,952	1,952 (NA)	NA
Cancer, stomach^¶^	58	56 (96.6)	3 (5.2)	943	897 (95.1)	46 (4.9)
Chronic hepatitis	2	2 (100.0)	0 (0.0)	42	36 (85.7)	6 (14.3)
Coronary heart disease	3,537	2,971 (84.0)	567 (16.0)	46,698	40,183 (86.0)	6,515 (14.0)
Degeneration of nervous system attributable to alcohol	145	118 (81.4)	27 (18.6)	2,617	2,030 (77.6)	587 (22.4)
Esophageal varices	112	77 (68.8)	34 (30.4)	2,414	1,711 (70.9)	703 (29.1)
Fetal alcohol syndrome	4	2 (50.0)	2 (50.0)	212	122 (57.5)	90 (42.5)
Fetus and newborn affected by maternal use of alcohol	1	1 (100.0)	0 (0.0)	76	76 (100.0)	0 (—)
Gallbladder disease	0	0 (—)	0 (—)	0	0 (—)	0 (—)
Gastroesophageal hemorrhage	31	20 (64.5)	10 (32.3)	517	359 (69.4)	157 (30.4)
Hypertension	3,584	1,638 (45.7)	1,946 (54.3)	50,016	26,021 (52.0)	23,994 (48.0)
Infant death, low birthweight**	2	1 (50.0)	1 (50.0)	133	69 (51.9)	65 (48.9)
Infant death, preterm birth**	44	24 (54.5)	19 (43.2)	3,410	1,845 (54.1)	1,565 (45.9)
Infant death, small for gestational age**	0	0 (—)	0 (—)	13	5 (38.5)	7 (53.8)
Liver cirrhosis, unspecified	9,801	5,696 (58.1)	4,105 (41.9)	197,875	114,580 (57.9)	83,295 (42.1)
Pancreatitis, acute	0	0 (—)	0 (—)	0	0 (—)	0 (—)
Pancreatitis, chronic	15	12 (80.0)	3 (20.0)	317	252 (79.5)	65 (20.5)
Pneumonia^††^	133	105 (78.9)	29 (21.8)	3,714	2,839 (76.4)	875 (23.6)
Portal hypertension	61	34 (55.7)	26 (42.6)	1,267	729 (57.5)	538 (42.5)
Stroke, hemorrhagic	938	565 (60.2)	374 (39.9)	14,497	8,856 (61.1)	5,641 (38.9)
Stroke, ischemic	342	243 (71.1)	100 (29.2)	3,867	2,837 (73.4)	1,030 (26.6)
Unprovoked seizures, epilepsy, or seizure disorder	134	112 (83.6)	22 (16.4)	3,987	3,352 (84.1	635 (15.9)
**Acute causes**	42,218	30,935 (73.3)	11,283 (26.7)	1,578,021	1,146,153 (72.6)	431,868 (27.4)
Air-space transport	75	64 (85.3)	11 (14.7)	2,268	1,867 (82.3)	401 (17.7)
Alcohol poisoning	2,288	1,735 (75.8)	553 (24.2)	76,224	56,511 (74.1)	19,713 (25.9)
Aspiration	255	141 (55.3)	114 (44.7)	4,765	2,695 (56.6)	2,070 (43.4)
Child maltreatment^§§^	148	87 (58.8)	61 (41.2)	11,000	6,294 (57.2)	4,706 (42.8)
Drowning	981	772 (78.7)	210 (21.4)	33,853	27,108 (80.1)	6,745 (19.9)
Fall injuries^¶¶^	2,645	1,873 (70.8)	772 (29.2)	70,815	49,887 (70.4)	20,927 (29.6)
Fire injuries	457	274 (60.0)	183 (40.0)	10,950	6,491 (59.3)	4,459 (40.7)
Firearm injuries	337	284 (84.3)	53 (15.7)	12,917	10,768 (83.4)	2,149 (16.6)
Homicide	5,306	4,267 (80.4)	1,039 (19.6)	230,047	187,052 (81.3)	42,995 (18.7)
Hypothermia	296	194 (65.5)	102 (34.5)	6,199	4,354 (70.2)	1,845 (29.8)
Motor-vehicle nontraffic crashes	190	144 (75.8)	47 (24.7)	5,588	4,249 (76.0)	1,339 (24.0)
Motor-vehicle traffic crashes***	7,092	5,522 (77.9)	1,570 (22.1)	323,610	245,447 (75.8)	78,163 (24.2)
Occupational and machine injuries	126	117 (92.9)	9 (7.1)	3,294	3,060 (92.9)	234 (7.1)
Other road vehicle crashes	170	137 (80.6)	33 (19.4)	5,632	4,473 (79.4)	1,159 (20.6)
Poisoning (not alcohol)	11,839	7,524 (63.6)	4,315 (36.4)	444,235	280,270 (63.1)	163,965 (36.9)
Suicide	9,899	7,711 (77.9)	2,189 (22.1)	332,791	252,674 (75.9)	80,117 (24.1)
Suicide by and exposure to alcohol	38	24 (63.2)	14 (36.8)	1,267	764 (60.3)	503 (39.7)
Water transport	75	65 (86.7)	9 (12.0)	2,566	2,189 (85.3)	377 (14.7)

**Abbreviation:** NA = not applicable.

* In the Alcohol-Related Disease Impact application (https://www.cdc.gov/ARDI), deaths attributable to excessive alcohol use include deaths from 1) conditions that are 100% alcohol-attributable, 2) deaths caused by acute conditions that involved binge drinking, and 3) deaths caused by chronic conditions that involved medium (>1 to ≤2 drinks of alcohol [women] or >2 to ≤4 drinks [men]) or high (>2 drinks of alcohol [women] or >4 drinks [men]) levels of average daily alcohol consumption.

^†^ Numbers might not sum to totals, and row percentages might not sum to 100% because of rounding.

^§^ Deaths calculated for the proportion of esophageal cancer deaths caused by squamous cell carcinoma only, based on the Surveillance, Epidemiology, and End Results data in 18 states (SEER18). https://seer.cancer.gov/.

^¶^ Deaths among those consuming high average daily levels of alcohol only.

** Alcohol consumption prevalence estimates calculated among women aged 18–44 years only.

^††^ Deaths among persons aged 20–64 years only because of the high number of deaths from pneumonia among persons aged ≥65 years that are not alcohol-related and the lack of relative risks that differ by age.

^§§^ Deaths among persons aged 0–14 years.

^¶¶^ Deaths among persons aged 15–69 years only because of the high number of deaths from falls among persons aged ≥70 years that are not alcohol-attributable and the lack of alcohol-attributable fractions that differ by age.

*** Deaths among persons of all ages. A blood alcohol concentration level of ≥0.08 g/dL is used for defining alcohol attribution for this condition.

**TABLE 2:** Annual average number of deaths and years of potential life lost from excessive alcohol use,* by state — United States, 2011–2015

Location	Alcohol-attributable deaths	Age-adjusted alcohol-attributable deaths per 100,000-population	Years of potential life lost	Years of potential life lost per 100,000-population	Years of potential life lost per alcohol-attributable death
**U.S. total**	**93,296**	**27.4**	**2,683,211**	**847.7**	**28.8**
Alabama	1,446	28.0	44,074	912.4	30.5
Alaska	292	29.4^†^	9,631	1,313.2	33.0
Arizona	2,594	37.0	74,450	1,120.9	28.7
Arkansas	892	28.3	26,512	896.2	29.7
California	10,811	26.9	299,336	779.1	27.7
Colorado	1,810	32.5	54,054	1,024.0	29.9
Connecticut	900	22.8	25,738	716.3	28.6
Delaware	271	19.3^†^	8,136	878.2	30.0
District of Columbia	207	26.4^†^	5,861	905.2	28.3
Florida	6,778	29.8	183,199	932.5	27.0
Georgia	2,556	24.7	75,681	756.3	29.6
Hawaii	348	17.1^†^	9,470	673.4	27.2
Idaho	491	29.5	14,037	868.3	28.6
Illinois	3,295	24.0	95,560	742.3	29.0
Indiana	1,900	27.4	56,502	860.2	29.7
Iowa	834	24.5	22,014	711.6	26.4
Kansas	750	24.7	22,152	765.7	29.5
Kentucky	1,524	32.3	45,422	1,032.9	29.8
Louisiana	1,523	31.5	47,217	1,020.9	31.0
Maine	424	18.8^†^	11,261	847.3	26.6
Maryland	1,453	22.9	43,804	738.6	30.1
Massachusetts	1,729	23.3	48,305	720.4	27.9
Michigan	3,123	28.9	89,332	902.3	28.6
Minnesota	1,333	22.7	36,537	674.2	27.4
Mississippi	913	29.3	27,950	935.4	30.6
Missouri	1,860	28.8	55,813	923.2	30.0
Montana	414	37.4	12,232	1,205.5	29.5
Nebraska	453	23.0	12,610	674.6	27.8
Nevada	1,037	34.6	29,604	1,057.8	28.5
New Hampshire	420	20.1^†^	11,364	858.2	27.1
New Jersey	1,967	20.3	57,455	645.2	29.2
New Mexico	1,129	52.3	34,424	1,651.7	30.5
New York	4,390	20.3	120,761	613.8	27.5
North Carolina	2,811	26.5	82,568	838.7	29.4
North Dakota	215	21.2^†^	6,352	880.2	29.5
Ohio	3,608	28.6	103,809	896.8	28.8
Oklahoma	1,465	36.4	43,597	1,132.5	29.8
Oregon	1,498	33.5	39,310	997.9	26.2
Pennsylvania	3,768	26.5	108,168	846.4	28.7
Rhode Island	337	20.5^†^	9,240	876.9	27.4
South Carolina	1,629	31.4	48,121	1,007.2	29.5
South Dakota	282	22.0^†^	8,608	1,020.9	30.5
Tennessee	2,102	30.0	62,325	958.9	29.7
Texas	7,097	26.9	213,553	804.7	30.1
Utah	68	26.1	21,803	751.0	31.9
Vermont	203	21.0^†^	5,074	809.8	25.0
Virginia	1,972	22.2	56,965	689.9	28.9
Washington	2,195	28.8	59,665	854.1	27.2
West Virginia	725	35.3	21,621	1,167.8	29.8
Wisconsin	1,722	27.2	47,374	825.0	27.5
Wyoming	236	27.1^†^	7,317	1,262.3	31.0

* In the Alcohol-Related Disease Impact application (https://www.cdc.gov/ARDI), deaths attributable to excessive alcohol use include deaths from 1) conditions that are 100% alcohol-attributable, 2) deaths caused by acute conditions that involved binge drinking, and 3) deaths caused by chronic conditions that involved medium (>1 to ≤2 drinks of alcohol [women] or >2 to ≤4 drinks [men]) or high (>2 drinks of alcohol [women] or >4 drinks [men]) levels of average daily alcohol consumption.

^†^ The estimate might be unreliable because of suppressed estimates of the number of alcohol-attributable deaths in two or more age groups, and estimates might not account for the total number of alcohol-attributable deaths in the state.

**FIGURE:**
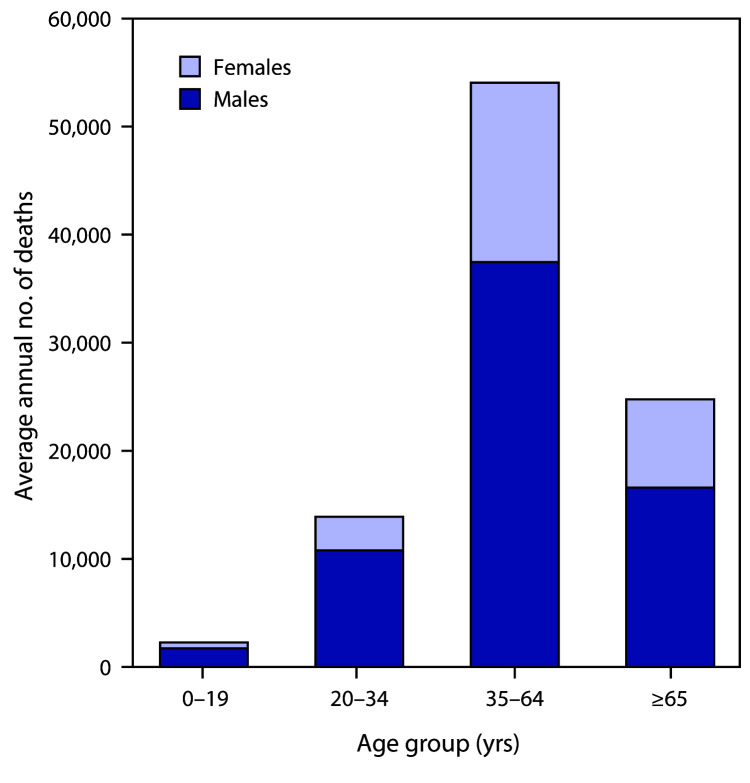
Average annual number of deaths attributable to excessive alcohol use,* by sex and age group — United States, 2011–2015 * In the Alcohol-Related Disease Impact application (https://www.cdc.gov/ARDI), deaths attributable to excessive alcohol use include deaths from 1) conditions that are 100% alcohol-attributable, 2) deaths caused by acute conditions that involved binge drinking, and 3) deaths caused by chronic conditions that involved medium (>1 to ≤2 drinks of alcohol [women] or >2 to ≤4 drinks [men]) or high (>2 drinks of alcohol [women] or >4 drinks [men]) levels of average daily alcohol consumption.

Alcoholic liver disease was the leading chronic cause of alcohol-attributable deaths overall (18,164) and among males (12,887) and females (5,277) (Table 1). Poisonings that involved another substance in addition to alcohol (e.g., drug overdoses) were the leading acute cause of alcohol-attributable deaths overall (11,839) and among females (4,315); suicide associated with excessive alcohol use was the leading acute cause of alcohol-attributable deaths among males (7,711). Conditions wholly attributable to alcohol accounted for 29,068 (31.2%) of all alcohol-attributable deaths and 762,241 (28.4%) of all YPLL.

The national average annual age-adjusted alcohol-attributable death rate was 27.4 per 100,000, and the YPLL per 100,000 was 847.7 (Table 2). The average annual number of alcohol-attributable deaths and YPLL varied across states, ranging from 203 alcohol-attributable deaths in Vermont to 10,811 in California, and from 5,074 YPLL in Vermont to 299,336 in California. Age-adjusted alcohol-attributable death rates among the 40 states with reliable estimates (excluding those with suppressed data where estimates might not account for all the alcohol-attributable deaths in the state) ranged from 20.3 per 100,000 in New Jersey and New York to 52.3 in New Mexico. YPLL per 100,000 ranged from 613.8 in New York to 1,651.7 in New Mexico.

## Discussion

Excessive alcohol use was responsible for approximately 93,000 deaths and 2.7 million YPLL annually in the United States during 2011–2015. This means that an average of 255 Americans die from excessive drinking every day, shortening their lives by an average of 29 years. The majority of these alcohol-attributable deaths involved males, and approximately four in five deaths involved adults aged ≥35 years. The number of alcohol-attributable deaths among adults aged ≥65 years was nearly double that among adults aged 20–34 years. Approximately one half of alcohol-attributable deaths were caused by chronic conditions, but acute alcohol-attributable deaths, all of which were caused by binge drinking, accounted for the majority of the YPLL from excessive drinking.

Little progress has been made in preventing deaths caused by excessive drinking; the average annual estimates of alcohol-attributable deaths and YPLL in this report are slightly higher than estimates for 2006–2010, and the age-adjusted alcohol-attributable death rates are similar (*5*), suggesting that excessive drinking remains a leading preventable cause of death and disability (*1*). From 2006–2010 (*5*) to 2011–2015, average annual deaths caused by alcohol dependence increased 14.2%, from 3,728 to 4,258, and deaths caused by alcoholic liver disease increased 23.6%, from 14,695 to 18,164. These findings are consistent with reported increasing trends in alcohol-induced deaths (e.g., deaths from conditions wholly attributable to alcohol) among adults aged ≥25 years,^†††^ including alcoholic liver disease,^§§§^ as well as with increases in per capita alcohol consumption during the past 2 decades.^¶¶¶^

Age-adjusted alcohol-attributable death rates varied approximately twofold across states, but deaths caused by excessive drinking were common across the country. The differences in alcohol-attributable death and YPLL rates in states might be partially explained by varying patterns of excessive alcohol use, particularly binge drinking, which is affected by state-level alcohol pricing and availability strategies (*6*) and differential access to medical care.

The findings in this report are subject to at least five limitations. First, the prevalence of alcohol consumption ascertained through the Behavioral Risk Factor Surveillance System is based on self-reported data, which substantially underestimates alcohol consumption (*7*). Second, these estimates are conservative, because former drinkers, some of whom might have died from alcohol-related conditions, are not included in the estimates of alcohol-attributable deaths and YPLL for partially alcohol-attributable causes of death. Third, direct alcohol-attributable fraction estimates for some chronic and acute conditions rely on data older than that of 2011–2015 (*4*) and might not accurately represent the proportion of excessive drinkers among persons who died of some conditions (e.g., drug overdoses) during that period. This emphasizes the importance of more timely information on alcohol involvement and various health conditions. Fourth, several conditions partially related to alcohol (e.g., tuberculosis, human immunodeficiency virus, and acquired immunodeficiency syndrome)**** are not included because published risk estimates were not available. Finally, the alcohol-attributable deaths and YPLL are based on alcohol-related conditions that were listed as the underlying (i.e., primary) cause of death, and not as a multiple cause of death, yielding conservative estimates.

The implementation of effective population-based strategies for preventing excessive drinking, such as those recommended by the Community Preventive Services Task Force (e.g., increasing alcohol taxes and regulating the number and concentration of alcohol outlets), could reduce alcohol-attributable deaths and YPLL. These strategies can complement other population-based prevention strategies that focus on health risk behaviors associated with excessive alcohol use, such as safer prescribing practices to reduce opioid misuse and overdoses (*8*,*9*) and alcohol-impaired driving interventions (*10*).

**Summary**

**What is already known about this topic?**

Excessive drinking is a leading cause of preventable death in the United States and is associated with numerous health and social problems.

**What is added by this report?**

During 2011–2015, excessive drinking was responsible for an average of 93,296 deaths (255 per day) and 2.7 million years of potential life lost (29 years lost per death, on average) in the United States each year.

**What are the implications for public health practice?**

Widespread implementation of prevention strategies, including those recommended by the Community Preventive Services Task Force (e.g., increasing alcohol taxes and regulating the number and concentration of places that sell alcohol) could help reduce deaths and years of potential life lost from excessive drinking.

## Footnotes

* https://www.cdc.gov/ARDI.

† https://www.thecommunityguide.org/topic/excessive-alcohol-consumption.

§ https://www.cdc.gov/alcohol/ardi/alcohol-related-icd-codes.html.

¶ https://www.cdc.gov/alcohol/ardi/methods.html.

** https://www.cdc.gov/brfss/.

†† https://www-fars.nhtsa.dot.gov/Crashes/CrashesAlcohol.aspx.

§§ Conditions that that are 100% alcohol-attributable include 13 chronic conditions (alcoholic psychosis, alcohol abuse, alcohol dependence syndrome, alcohol polyneuropathy, degeneration of the nervous system caused by alcohol use, alcoholic myopathy, alcohol cardiomyopathy, alcoholic gastritis, alcoholic liver disease, alcohol-induced acute pancreatitis, alcohol-induced chronic pancreatitis, fetal alcohol syndrome, and fetus and newborn affected by maternal use of alcohol) and two acute conditions (suicide by and exposure to alcohol and alcohol poisoning).

¶¶ https://www.cdc.gov/mmwr/preview/mmwrhtml/00001773.htm.

*** https://www.cdc.gov/nchs/data/statnt/statnt20.pdf.

††† https://www.cdc.gov/mmwr/volumes/68/wr/mm6833a5.htm.

§§§ https://pubs.niaaa.nih.gov/publications/surveillance111/Cirr15.htm.

¶¶¶ https://pubs.niaaa.nih.gov/publications/surveillance110/CONS16.htm.

**** https://apps.who.int/iris/bitstream/handle/10665/274603/9789241565639-eng.pdf?ua.
