# Correction and Republication: Deaths and Years of Potential Life Lost From Excessive Alcohol Use — United States, 2011–2015

**DOI:** 10.15585/mmwr.mm6939a5

**Published:** 2020-10-02

On July 31, 2020, *MMWR* published “Deaths and Years of Potential Life Lost From Excessive Alcohol Use — United States, 2011–2015” (*1*). On August 19, 2020, the authors informed *MMWR* that some results were inaccurate because of a data input error that occurred during an update to the Alcohol-Related Disease Impact application (*2*) used in the study. This error resulted in an overall underestimate of average annual alcohol-attributable deaths by 1,862 (from 93,296 to 95,158) and years of potential life lost by 79,844 (from 2,683,211 to 2,763,055) for the United States during 2011–2015. On September 3, 2020, corrections were made in the online Alcohol-Related Disease Impact application to the alcohol-attributable fractions for five acute causes of death: drownings, fall injuries, fire injuries, firearm injuries, and homicide. The authors have corrected the *MMWR* report accordingly and confirmed that the interpretation and the conclusions of the original report were not affected by these corrections. In accordance with December 2017 guidance from the International Committee of Medical Journal Editors (*3*), *MMWR* is republishing the report (*4*). The republished report includes the original report with clearly marked corrections in supplementary materials.
